# Supplementary material for: Development and validation of an end stage kidney disease awareness survey: Item difficulty and discrimination indices
Source: PLoS One. 2022 Sep 9;17(9):e0269488. doi: 10.1371/journal.pone.0269488 (PMC9462569; doi:10.1371/journal.pone.0269488)
Supplement: S1 File — (DOCX) [file pone.0269488.s002.docx]

Item-Level Analysis from R’s psychometric package: Full Scale, General Kidney Knowledge Domain

|  | Item SD | Item Correlation  with Domain Score | Item Correlation  with Total Score  (scored without item) | Difficulty | Discrimination | Item Reliability | Item Reliability  (scored without item) |
| --- | --- | --- | --- | --- | --- | --- | --- |
| Q1 | 0.37 | 0.65 | 0.55 | 0.84 | 0.42 | 0.24 | 0.20 |
| Q2 | 0.41 | 0.65 | 0.54 | 0.79 | 0.47 | 0.27 | 0.22 |
| Q3 | 0.49 | 0.57 | 0.41 | 0.60 | 0.69 | 0.28 | 0.20 |
| Q4 | 0.39 | 0.37 | 0.23 | 0.19 | 0.36 | 0.14 | 0.09 |
| Q7 | 0.33 | 0.60 | 0.51 | 0.88 | 0.28 | 0.19 | 0.16 |
| Q8 | 0.49 | 0.56 | 0.40 | 0.59 | 0.56 | 0.27 | 0.20 |
| Q9 | 0.37 | 0.82 | 0.76 | 0.84 | 0.47 | 0.30 | 0.28 |
| Q14 | 0.45 | 0.68 | 0.57 | 0.73 | 0.58 | 0.30 | 0.25 |
| Q15 | 0.40 | 0.69 | 0.59 | 0.81 | 0.50 | 0.27 | 0.23 |
| Q39 | 0.50 | 0.48 | 0.31 | 0.48 | 0.64 | 0.24 | 0.15 |

SD: Standard Deviation

Cronbach’s alpha = .80

Item-Level Analysis from R’s psychometric package: Full Scale, Chronic Kidney Disease (CKD) Knowledge Domain

|  | Item SD | Item Correlation  with Domain Score | Item Correlation  with Total Score  (scored without item) | Difficulty | Discrimination | Item Reliability | Item Reliability  (scored without item) |
| --- | --- | --- | --- | --- | --- | --- | --- |
| Q5 | 0.50 | 0.61 | 0.54 | 0.56 | 0.75 | 0.30 | 0.27 |
| Q6 | 0.50 | 0.61 | 0.53 | 0.44 | 0.72 | 0.30 | 0.26 |
| Q10 | 0.48 | 0.51 | 0.42 | 0.65 | 0.42 | 0.24 | 0.20 |
| Q11 | 0.50 | 0.59 | 0.51 | 0.56 | 0.69 | 0.29 | 0.25 |
| Q12 | 0.50 | 0.60 | 0.51 | 0.51 | 0.67 | 0.30 | 0.26 |
| Q13 | 0.50 | 0.56 | 0.48 | 0.42 | 0.58 | 0.28 | 0.24 |
| Q16 | 0.50 | 0.48 | 0.38 | 0.44 | 0.53 | 0.24 | 0.19 |
| Q17 | 0.50 | 0.58 | 0.49 | 0.46 | 0.61 | 0.29 | 0.25 |
| Q18 | 0.45 | 0.44 | 0.35 | 0.71 | 0.33 | 0.20 | 0.16 |
| Q19 | 0.36 | 0.43 | 0.36 | 0.15 | 0.33 | 0.15 | 0.13 |
| Q20 | 0.28 | 0.28 | 0.22 | 0.08 | 0.17 | 0.08 | 0.06 |
| Q21 | 0.28 | 0.12 | 0.06 | 0.08 | 0.08 | 0.03 | 0.02 |
| Q36 | 0.49 | 0.60 | 0.52 | 0.60 | 0.64 | 0.29 | 0.25 |
| Q37 | 0.45 | 0.50 | 0.42 | 0.27 | 0.53 | 0.22 | 0.19 |
| Q38 | 0.47 | 0.60 | 0.52 | 0.67 | 0.61 | 0.28 | 0.25 |
| Q40 | 0.42 | 0.50 | 0.43 | 0.22 | 0.50 | 0.21 | 0.18 |
| Q41 | 0.40 | 0.36 | 0.28 | 0.19 | 0.33 | 0.14 | 0.11 |
| Q42 | 0.29 | 0.26 | 0.19 | 0.09 | 0.17 | 0.07 | 0.06 |
| Q43 | 0.23 | 0.17 | 0.12 | 0.06 | 0.06 | 0.04 | 0.03 |
| Q44 | 0.50 | 0.64 | 0.57 | 0.58 | 0.75 | 0.32 | 0.28 |
| Q45 | 0.41 | 0.24 | 0.15 | 0.21 | 0.22 | 0.10 | 0.06 |

SD: Standard Deviation

Cronbach’s alpha = .83

Item-Level Analysis from R’s psychometric package: Full Scale, End Stage Kidney Disease (ESKD) Knowledge Domain

|  | Item SD | Item Correlation  with Domain Score | Item Correlation  with Total Score  (scored without item) | Difficulty | Discrimination | Item Reliability | Item Reliability  (scored without item) |
| --- | --- | --- | --- | --- | --- | --- | --- |
| Q22 | 0.50 | 0.47 | 0.34 | 0.50 | 0.56 | 0.23 | 0.17 |
| Q23 | 0.50 | 0.52 | 0.41 | 0.44 | 0.58 | 0.26 | 0.20 |
| Q24 | 0.50 | 0.54 | 0.43 | 0.43 | 0.56 | 0.27 | 0.21 |
| Q25 | 0.50 | 0.70 | 0.62 | 0.44 | 0.81 | 0.35 | 0.31 |
| Q26 | 0.50 | 0.72 | 0.64 | 0.44 | 0.86 | 0.36 | 0.32 |
| Q27 | 0.48 | 0.55 | 0.45 | 0.34 | 0.58 | 0.26 | 0.21 |
| Q28 | 0.46 | 0.60 | 0.51 | 0.30 | 0.72 | 0.28 | 0.23 |
| Q29 | 0.49 | 0.71 | 0.64 | 0.39 | 0.83 | 0.35 | 0.31 |
| Q30 | 0.43 | 0.56 | 0.47 | 0.24 | 0.53 | 0.24 | 0.20 |
| Q31 | 0.40 | 0.48 | 0.39 | 0.19 | 0.47 | 0.19 | 0.15 |
| Q32 | 0.49 | 0.46 | 0.34 | 0.37 | 0.47 | 0.22 | 0.17 |
| Q33 | 0.50 | 0.66 | 0.57 | 0.52 | 0.75 | 0.33 | 0.29 |
| Q34 | 0.46 | 0.42 | 0.30 | 0.31 | 0.44 | 0.19 | 0.14 |
| Q35 | 0.19 | 0.20 | 0.14 | 0.04 | 0.08 | 0.04 | 0.03 |

SD: Standard Deviation

Cronbach’s alpha = .82

Item-Level Analysis from R’s psychometric package: Full Scale, Total Score

|  | Item SD | Item Correlation  with Total Score | Item Correlation  with Total Score  (scored without item) | Difficulty | Discrimination | Item Reliability | Item Reliability  (scored without item) |
| --- | --- | --- | --- | --- | --- | --- | --- |
| Q1 | 0.37 | 0.54 | 0.51 | 0.84 | 0.39 | 0.20 | 0.18 |
| Q2 | 0.41 | 0.50 | 0.46 | 0.79 | 0.42 | 0.20 | 0.19 |
| Q3 | 0.49 | 0.37 | 0.32 | 0.60 | 0.31 | 0.18 | 0.16 |
| Q4 | 0.39 | 0.16 | 0.12 | 0.19 | 0.08 | 0.06 | 0.05 |
| Q5 | 0.50 | 0.64 | 0.60 | 0.56 | 0.78 | 0.32 | 0.30 |
| Q6 | 0.50 | 0.57 | 0.53 | 0.44 | 0.67 | 0.28 | 0.26 |
| Q7 | 0.33 | 0.50 | 0.47 | 0.88 | 0.28 | 0.16 | 0.15 |
| Q8 | 0.49 | 0.42 | 0.38 | 0.59 | 0.44 | 0.21 | 0.19 |
| Q9 | 0.37 | 0.68 | 0.66 | 0.84 | 0.44 | 0.25 | 0.24 |
| Q10 | 0.48 | 0.52 | 0.48 | 0.65 | 0.47 | 0.25 | 0.23 |
| Q11 | 0.50 | 0.56 | 0.53 | 0.56 | 0.64 | 0.28 | 0.26 |
| Q12 | 0.50 | 0.57 | 0.53 | 0.51 | 0.67 | 0.28 | 0.26 |
| Q13 | 0.50 | 0.53 | 0.49 | 0.42 | 0.58 | 0.26 | 0.24 |
| Q14 | 0.45 | 0.57 | 0.53 | 0.73 | 0.53 | 0.25 | 0.24 |
| Q15 | 0.40 | 0.56 | 0.53 | 0.81 | 0.42 | 0.22 | 0.21 |
| Q16 | 0.50 | 0.44 | 0.39 | 0.44 | 0.47 | 0.22 | 0.19 |
| Q17 | 0.50 | 0.53 | 0.49 | 0.46 | 0.56 | 0.26 | 0.24 |
| Q18 | 0.45 | 0.44 | 0.40 | 0.71 | 0.39 | 0.20 | 0.18 |
| Q19 | 0.36 | 0.38 | 0.34 | 0.15 | 0.31 | 0.13 | 0.12 |
| Q20 | 0.28 | 0.15 | 0.12 | 0.08 | 0.06 | 0.04 | 0.03 |
| Q21 | 0.28 | 0.13 | 0.10 | 0.08 | 0.08 | 0.03 | 0.03 |
| Q22 | 0.50 | 0.41 | 0.36 | 0.50 | 0.36 | 0.20 | 0.18 |
| Q23 | 0.50 | 0.47 | 0.42 | 0.44 | 0.53 | 0.23 | 0.21 |
| Q24 | 0.50 | 0.49 | 0.44 | 0.43 | 0.58 | 0.24 | 0.22 |
| Q25 | 0.50 | 0.58 | 0.55 | 0.44 | 0.72 | 0.29 | 0.27 |
| Q26 | 0.50 | 0.59 | 0.55 | 0.44 | 0.72 | 0.29 | 0.27 |
| Q27 | 0.48 | 0.51 | 0.47 | 0.34 | 0.58 | 0.24 | 0.22 |
| Q28 | 0.46 | 0.47 | 0.43 | 0.30 | 0.56 | 0.21 | 0.19 |
| Q29 | 0.49 | 0.61 | 0.58 | 0.39 | 0.78 | 0.30 | 0.28 |
| Q30 | 0.43 | 0.42 | 0.38 | 0.24 | 0.42 | 0.18 | 0.16 |
| Q31 | 0.40 | 0.36 | 0.32 | 0.19 | 0.33 | 0.14 | 0.13 |
| Q32 | 0.49 | 0.49 | 0.45 | 0.37 | 0.56 | 0.24 | 0.22 |
| Q33 | 0.50 | 0.66 | 0.62 | 0.52 | 0.81 | 0.33 | 0.31 |
| Q34 | 0.46 | 0.41 | 0.36 | 0.31 | 0.39 | 0.19 | 0.17 |
| Q35 | 0.19 | 0.14 | 0.12 | 0.04 | 0.06 | 0.03 | 0.02 |
| Q36 | 0.49 | 0.55 | 0.51 | 0.60 | 0.61 | 0.27 | 0.25 |
| Q37 | 0.45 | 0.40 | 0.36 | 0.27 | 0.42 | 0.18 | 0.16 |
| Q38 | 0.47 | 0.52 | 0.48 | 0.67 | 0.50 | 0.25 | 0.23 |
| Q39 | 0.50 | 0.46 | 0.42 | 0.48 | 0.44 | 0.23 | 0.21 |
| Q40 | 0.42 | 0.44 | 0.40 | 0.22 | 0.42 | 0.18 | 0.17 |
| Q41 | 0.40 | 0.38 | 0.34 | 0.19 | 0.31 | 0.15 | 0.14 |
| Q42 | 0.29 | 0.16 | 0.13 | 0.09 | 0.08 | 0.05 | 0.04 |
| Q43 | 0.23 | 0.18 | 0.15 | 0.06 | 0.08 | 0.04 | 0.03 |
| Q44 | 0.50 | 0.64 | 0.60 | 0.58 | 0.75 | 0.31 | 0.30 |
| Q45 | 0.41 | 0.21 | 0.17 | 0.21 | 0.11 | 0.09 | 0.07 |

SD: Standard Deviation

Cronbach’s alpha = .92

Item-Level Analysis from R’s psychometric package: Reduced Scale, General Kidney Knowledge Domain

|  | Item SD | Item Correlation  with Domain Score | Item Correlation  with Total Score  (scored without item) | Difficulty | Discrimination | Item Reliability | Item Reliability  (scored without item) |
| --- | --- | --- | --- | --- | --- | --- | --- |
| Q2 | 0.41 | 0.59 | 0.35 | 0.79 | 0.44 | 0.24 | 0.14 |
| Q3 | 0.49 | 0.67 | 0.41 | 0.60 | 0.69 | 0.33 | 0.20 |
| Q8 | 0.49 | 0.62 | 0.34 | 0.59 | 0.64 | 0.31 | 0.17 |
| Q14 | 0.45 | 0.68 | 0.45 | 0.73 | 0.64 | 0.30 | 0.20 |
| Q39 | 0.50 | 0.54 | 0.22 | 0.48 | 0.53 | 0.27 | 0.11 |

SD: Standard Deviation

Cronbach’s alpha = .59

Item-Level Analysis from R’s psychometric package: Reduced Scale, Chronic Kidney Disease (CKD) Knowledge Domain

|  | Item SD | Item Correlation  with Domain Score | Item Correlation  with Total Score  (scored without item) | Difficulty | Discrimination | Item Reliability | Item Reliability  (scored without item) |
| --- | --- | --- | --- | --- | --- | --- | --- |
| Q5 | 0.50 | 0.65 | 0.56 | 0.56 | 0.75 | 0.32 | 0.28 |
| Q6 | 0.50 | 0.64 | 0.55 | 0.44 | 0.75 | 0.32 | 0.27 |
| Q10 | 0.48 | 0.55 | 0.45 | 0.65 | 0.61 | 0.26 | 0.22 |
| Q11 | 0.50 | 0.62 | 0.53 | 0.56 | 0.72 | 0.31 | 0.26 |
| Q12 | 0.50 | 0.61 | 0.52 | 0.51 | 0.67 | 0.31 | 0.26 |
| Q13 | 0.50 | 0.57 | 0.47 | 0.42 | 0.61 | 0.28 | 0.23 |
| Q16 | 0.50 | 0.47 | 0.36 | 0.44 | 0.53 | 0.23 | 0.18 |
| Q17 | 0.50 | 0.62 | 0.53 | 0.46 | 0.72 | 0.31 | 0.27 |
| Q18 | 0.45 | 0.47 | 0.37 | 0.71 | 0.42 | 0.21 | 0.17 |
| Q36 | 0.49 | 0.58 | 0.48 | 0.60 | 0.64 | 0.28 | 0.24 |
| Q37 | 0.45 | 0.51 | 0.41 | 0.27 | 0.50 | 0.22 | 0.18 |
| Q38 | 0.47 | 0.61 | 0.52 | 0.67 | 0.61 | 0.29 | 0.25 |
| Q40 | 0.42 | 0.47 | 0.38 | 0.22 | 0.42 | 0.19 | 0.16 |
| Q44 | 0.50 | 0.65 | 0.57 | 0.58 | 0.69 | 0.32 | 0.28 |

SD: Standard Deviation

Cronbach’s alpha = .84

Item-Level Analysis from R’s psychometric package: Reduced Scale, End Stage Kidney Disease (ESKD) Knowledge Domain

|  | Item SD | Item Correlation  with Domain Score | Item Correlation  with Total Score  (scored without item) | Difficulty | Discrimination | Item Reliability | Item Reliability  (scored without item) |
| --- | --- | --- | --- | --- | --- | --- | --- |
| Q22 | 0.50 | 0.47 | 0.35 | 0.50 | 0.58 | 0.23 | 0.17 |
| Q23 | 0.50 | 0.52 | 0.41 | 0.44 | 0.58 | 0.26 | 0.20 |
| Q24 | 0.50 | 0.54 | 0.43 | 0.43 | 0.56 | 0.27 | 0.21 |
| Q25 | 0.50 | 0.70 | 0.62 | 0.44 | 0.81 | 0.35 | 0.31 |
| Q26 | 0.50 | 0.72 | 0.64 | 0.44 | 0.86 | 0.36 | 0.32 |
| Q27 | 0.48 | 0.55 | 0.45 | 0.34 | 0.58 | 0.26 | 0.21 |
| Q28 | 0.46 | 0.60 | 0.51 | 0.30 | 0.69 | 0.27 | 0.23 |
| Q29 | 0.49 | 0.72 | 0.64 | 0.39 | 0.83 | 0.35 | 0.31 |
| Q30 | 0.43 | 0.55 | 0.46 | 0.24 | 0.56 | 0.24 | 0.20 |
| Q32 | 0.49 | 0.46 | 0.34 | 0.37 | 0.44 | 0.22 | 0.16 |
| Q33 | 0.50 | 0.67 | 0.57 | 0.52 | 0.75 | 0.33 | 0.29 |
| Q34 | 0.46 | 0.42 | 0.31 | 0.31 | 0.44 | 0.20 | 0.14 |
| Q31 | 0.40 | 0.48 | 0.38 | 0.19 | 0.47 | 0.19 | 0.15 |

SD: Standard Deviation

Cronbach’s alpha = .83

Item-Level Analysis from R’s psychometric package: Reduced Scale, Total Score

|  | Item SD | Item Correlation  with Total Score | Item Correlation  with Total Score  (scored without item) | Difficulty | Discrimination | Item Reliability | Item Reliability  (scored without item) |
| --- | --- | --- | --- | --- | --- | --- | --- |
| Q2 | 0.41 | 0.48 | 0.44 | 0.79 | 0.42 | 0.2 | 0.18 |
| Q3 | 0.49 | 0.34 | 0.29 | 0.6 | 0.28 | 0.17 | 0.14 |
| Q5 | 0.5 | 0.64 | 0.6 | 0.56 | 0.78 | 0.32 | 0.3 |
| Q6 | 0.5 | 0.58 | 0.54 | 0.44 | 0.67 | 0.29 | 0.27 |
| Q8 | 0.49 | 0.42 | 0.37 | 0.59 | 0.42 | 0.21 | 0.18 |
| Q10 | 0.48 | 0.51 | 0.46 | 0.65 | 0.5 | 0.24 | 0.22 |
| Q11 | 0.5 | 0.58 | 0.53 | 0.56 | 0.58 | 0.29 | 0.27 |
| Q12 | 0.5 | 0.57 | 0.52 | 0.51 | 0.64 | 0.28 | 0.26 |
| Q13 | 0.5 | 0.54 | 0.49 | 0.42 | 0.61 | 0.26 | 0.24 |
| Q14 | 0.45 | 0.56 | 0.52 | 0.73 | 0.53 | 0.25 | 0.23 |
| Q16 | 0.5 | 0.44 | 0.39 | 0.44 | 0.44 | 0.22 | 0.19 |
| Q17 | 0.5 | 0.55 | 0.5 | 0.46 | 0.64 | 0.27 | 0.25 |
| Q18 | 0.45 | 0.44 | 0.39 | 0.71 | 0.42 | 0.2 | 0.18 |
| Q22 | 0.5 | 0.41 | 0.35 | 0.5 | 0.39 | 0.2 | 0.18 |
| Q23 | 0.5 | 0.47 | 0.42 | 0.44 | 0.5 | 0.23 | 0.21 |
| Q24 | 0.5 | 0.48 | 0.43 | 0.43 | 0.58 | 0.24 | 0.21 |
| Q25 | 0.5 | 0.61 | 0.57 | 0.44 | 0.78 | 0.3 | 0.28 |
| Q26 | 0.5 | 0.61 | 0.56 | 0.44 | 0.75 | 0.3 | 0.28 |
| Q27 | 0.48 | 0.51 | 0.46 | 0.34 | 0.56 | 0.24 | 0.22 |
| Q28 | 0.46 | 0.5 | 0.45 | 0.3 | 0.53 | 0.23 | 0.21 |
| Q29 | 0.49 | 0.63 | 0.59 | 0.39 | 0.75 | 0.31 | 0.29 |
| Q30 | 0.43 | 0.42 | 0.37 | 0.24 | 0.44 | 0.18 | 0.16 |
| Q32 | 0.49 | 0.5 | 0.45 | 0.37 | 0.56 | 0.24 | 0.22 |
| Q33 | 0.5 | 0.67 | 0.63 | 0.52 | 0.81 | 0.33 | 0.32 |
| Q34 | 0.46 | 0.41 | 0.36 | 0.31 | 0.36 | 0.19 | 0.16 |
| Q36 | 0.49 | 0.54 | 0.5 | 0.6 | 0.64 | 0.27 | 0.24 |
| Q37 | 0.45 | 0.43 | 0.39 | 0.27 | 0.42 | 0.19 | 0.17 |
| Q38 | 0.47 | 0.52 | 0.48 | 0.67 | 0.5 | 0.25 | 0.23 |
| Q39 | 0.5 | 0.45 | 0.4 | 0.48 | 0.47 | 0.22 | 0.2 |
| Q40 | 0.42 | 0.43 | 0.39 | 0.22 | 0.42 | 0.18 | 0.16 |
| Q44 | 0.5 | 0.65 | 0.61 | 0.58 | 0.72 | 0.32 | 0.3 |
| Q31 | 0.4 | 0.37 | 0.33 | 0.19 | 0.36 | 0.15 | 0.13 |

SD: Standard Deviation

Cronbach’s alpha = .91
