# Supplementary material for: Development and validation of an end stage kidney disease awareness survey: Item difficulty and discrimination indices
Source: PLoS One. 2022 Sep 9;17(9):e0269488. doi: 10.1371/journal.pone.0269488 (PMC9462569; doi:10.1371/journal.pone.0269488)
Supplement: S2 File — (DOCX) [file pone.0269488.s003.docx]

**Shukla et. al. Kidney Failure Assessment Questionnaire**

**Full Scale**

(Please circle the best answer)

1. **What do the kidneys do? [Domain: General Kidney Knowledge; G]**
   1. Check sugar level in blood
   2. Remove waste from blood
   3. Carry blood to the muscles
   4. Soak up vitamins from food
2. **What is the kidney system called? [G]**
   1. Pulmonary system
   2. Endocrine system
   3. Nervous system
   4. Renal system
3. **What should people with failing kidneys know? [G]**
   1. Do not take over the counter pain meds
   2. Do not go out to crowded places
   3. Do not make your body tired
   4. Do not take over the counter nausea meds
4. **What values in the blood tests can estimate the level of kidney function for an individual patient? [G]**
   1. Blood urea nitrogen
   2. serum creatinine
   3. Potassium level
   4. both a & b
   5. a, b & c
5. **When your kidneys do not work appropriately for a long time (example: more than 3 months), it is called chronic kidney disease (CKD). How many stages of chronic kidney disease (CKD) are there? [CKD Knowledge; CKD]**
   1. 3
   2. 5
   3. 7
   4. 9
6. **Kidney patients often need dialysis (way to clean the blood) when: [CKD]**
   1. eGFR less than 60 or stage 3
   2. eGFR less than 45 or stage 3
   3. eGFR less than 30 or stage 4
   4. eGFR less than 15 or stage 5
7. **Which one of the following is one of the most common causes of kidney failure worldwide? [G]**
   1. Diabetes (high blood sugar)
   2. Glaucoma
   3. Acid reflux
   4. Asthma
8. **A patient is producing normal amount of urine, has no swelling and has no symptoms: These mean that the kidneys must be functioning well? [G]**
   1. true
   2. false
9. **Anemia (low iron) is what type of disease? [G]**
   1. blood disease with low hemoglobin
   2. heart disease with chest pain
   3. lung disease with cough
   4. bone disease with pain
10. **As the kidneys stop working well, what usually happens to creatinine level? [CKD]**
    1. rises
    2. stays the same
    3. lowers
    4. none of the above
11. **As the kidneys stop working well, what usually can happens to potassium levels? [CKD]**
    1. rises
    2. stays the same
    3. lowers
    4. none of the above
12. **As the kidney function gets worse, what happens to the estimated glomerular filtration rate values (eGFR)? [CKD]**
    1. rises
    2. stays the same
    3. lowers
    4. none of the above
13. **As the kidneys get worse, what usually happens to the hemoglobin level? [CKD]**
    1. rises
    2. stays the same
    3. lowers
    4. none of the above
14. **Besides the hormone erythropoietin, what other medication is often needed to treat anemia? [G]**
    1. Iron
    2. Ibuprofen
    3. Tylenol
    4. Vitamin A
15. **What do you call a kidney doctor? [G]**
    1. neurologist
    2. cardiologist
    3. nephrologist
    4. dermatologist
16. **Failing kidneys can cause this vitamin to not work the way it should. [CKD]**
    1. Vitamin A
    2. Vitamin C
    3. Vitamin D
    4. Vitamin E
17. **High potassium levels in blood are usually not symptomatic. Then, should one worry about the potassium intake when they have CKD? [CKD]**
    1. It can cause sudden hearth rhythm problems and can cause death
    2. It can increase the blood pressure
    3. It can be associated with sluggish mentation
    4. No, potassium levels are not worrisome since they are harmless
18. **Low levels of potassium are safe. [CKD]**
    1. True
    2. False
19. **The best way to control the blood levels of phosphorus is/are: [CKD]**
    1. taking medications to reduce the phosphorus absorption
    2. limit dietary intake by reducing certain foods and drinks
    3. exercise
    4. a & b
    5. a, b & c
20. **Which medications are used to lower phosphorus? [CKD]**
    1. calcium salts
    2. sevelamer
    3. Vitamin D
    4. a & b
    5. a, b, and c
21. **Some of the common foods such as milk, soda and red meats are high in phosphorus content. To lower the phosphorus in your body, you should take your meds. When is the best time to take your phosphorus lowering meds? [CKD]**
    1. one hour prior to meal
    2. just before meal
    3. during (in between) meals while eating food.
    4. just after meals
22. **What is the best type of kidney replacement therapy for people with failing kidneys? [End Stage Kidney Disease Knowledge; ESKD]**
    1. kidney transplantation
    2. neither dialysis nor transplantation
    3. hemodialysis (blood dialysis)
    4. peritoneal dialysis (dialysis through abdomen)
23. **What type of dialysis has the best results? [ESKD]**
    1. in center hemodialysis
    2. peritoneal dialysis
    3. home hemodialysis
    4. all dialysis are equal in terms of outcome, it's about individual choice.
24. **Which type of dialysis makes it easier to travel? [ESKD]**
    1. in center dialysis
    2. home dialysis
    3. both
    4. none
25. **Peritoneal dialysis is [ESKD]**
    1. dialysis with a tube in the belly
    2. blood dialysis therapy needed 3 times a week that uses needles or bloodline
    3. blood dialysis therapy 6 days a week that uses needles or bloodline
    4. all of the above
26. **In center hemodialysis is [ESKD]**
    1. dialysis with a tube in the belly
    2. blood dialysis therapy needed 3 times a week that uses needles or bloodline
    3. blood dialysis therapy 6 days a week that uses needles or bloodline
    4. all of the above
27. **AV (arteriovenous) fistula used for hemodialysis is: [ESKD]**
    1. a surgical linking of an artery and vein
    2. a tube under the skin on your arm
    3. a tube coming out of your neck
    4. a tube coming from your belly
28. **AV (arteriovenous) graft used for hemodialysis is: [ESKD]**
    1. a surgical linking of an artery and vein
    2. a tube under the skin linking an artery and a vein
    3. a tube coming out of your neck
    4. a tube coming from your belly
29. **In center hemodialysis is done: [ESKD]**
    1. 3 times weekly for 2 hours
    2. 3 times weekly for 4 hours
    3. 5 times weekly for 2 hours
    4. 5 times weekly for 4 hours
30. **When is a good time to place a peritoneal dialysis tube? [ESKD]**
    1. around 2 - 4 weeks before the dialysis is needed
    2. after starting in center hemodialysis
    3. up to 6 months before dialysis is needed
    4. during training for peritoneal dialysis
31. **When is a good time to place a fistula to be used for hemodialysis access? [ESKD]**
    1. just before starting the first dialysis
    2. after dialysis has been started
    3. up to 6 months before dialysis is needed
    4. 2 weeks before dialysis is needed
32. **You can be placed on the list for a kidney transplant when your kidney function gets below: [ESKD]**
    1. 50 %
    2. 40 %
    3. 30 %
    4. 20 %
33. **End Stage Renal Disease (ESRD) and initiation of dialysis is a major disability and requires for people to quit their jobs when they start dialysis. [ESKD]**
    1. True
    2. False
34. **If you get Medicare and you decide to do a home dialysis, when does your Medicare coverage begin? [ESKD]**
    1. the month you begin dialysis
    2. after one month
    3. after three months
    4. after 6 months
35. **If you get Medicare and decide to do in center hemodialysis, when does your Medicare coverage begin? [ESKD]**
    1. the month you begin dialysis
    2. after one month
    3. after three months
    4. after 6 months
36. **Which vitamin is an important part of healthy bones in CKD? [CKD]**
    1. Vitamin A
    2. Vitamin B
    3. Vitamin C
    4. Vitamin D
37. **When are phosphate binders given? [CKD]**
    1. when phosphorus is too low
    2. when phosphorus is too high
    3. when potassium is too low
    4. when potassium is too high
38. **Smokers have more problems with failing kidneys than non-smokers. [CKD]**
    1. True
    2. False
39. **Foamy urine may suggest that you are losing _____________ in your urine. [G]**
    1. potassium
    2. protein
    3. creatinine
    4. urea
40. **High phosphorus levels can cause: [CKD]**
    1. weight loss
    2. hardening of blood vessel walls
    3. weakening of the pancreas
    4. liver damage
41. **As your kidney disease gets worse, what builds up in your blood? [CKD]**
    1. acid
    2. alkali
    3. proteins
    4. bicarbonate
42. **Erythropoietin (EPO) is a hormone made by kidneys that helps individuals’ body to make red blood cells. Therapy with EPO is usually targeted to achieve which of the following? [CKD]**
    1. a near normal hemoglobin at about 12-14gm/dl
    2. maintain low hemoglobin at about 9-11gm/dl
    3. maintain low hemoglobin at about 7-9gm/dl
    4. EPO is dangerous and thus should never be used
43. **High parathyroid hormone (PTH) levels are detrimental to the health of bones and heart. In addition to keeping the phosphorus under control which of the following class of medication may be needed to treat high PTH levels? [CKD]**
    1. anti-hypertensive medications
    2. erythropoietin therapy
    3. Vitamin D therapy
    4. all of the above
44. **Chronic kidney disease (CKD) increases the risk of heart attack [CKD]**
    1. True
    2. False
45. **Which medications can help lower protein in urine? [CKD]**
    1. some blood pressure medications (Lisinopril and Losartan)
    2. an active form of Vitamin D (paricalcitol)
    3. some diuretics (spironolactone and eplerenone)
    4. all of the above
