# Supplementary material for: Development and validation of an end stage kidney disease awareness survey: Item difficulty and discrimination indices
Source: PLoS One. 2022 Sep 9;17(9):e0269488. doi: 10.1371/journal.pone.0269488 (PMC9462569; doi:10.1371/journal.pone.0269488)
Supplement: Attachment — Submitted filename: Response to PlosOne Review.3.21.docx [file pone.0269488.s005.docx]

Date: March 21, 2022

Dear Editor,

Thank you for the review of our manuscript titled “Development and Validation of an End Stage Kidney Disease Awareness Survey: Item Difficulty and Discrimination Indices".

We appreciate your feedback and have revised the manuscript to ensure that the stated concerns are addressed.

We would be happy to provide any further information desired.

Thank you,

Ashutosh M Shukla, MD,

On behalf of all co-authors.

**Reviewer #1:**

The authors did a great work creating such an excellent questionnaire to assess ESKD awareness among kidney patients. There are, however, some points to be revised.

1. *The full-scale questionnaire included questions that are currently under a strong debate among the nephrology community. The use of word renal and nephrology are starting to vanish. Q2 and Q15 asked about these items in particular. While there is an international movement towards the use of kidney instead of renal to be closer to the patient understanding. For example, we moved from acute renal failure to acute kidney injury, and from end-stage renal disease (ESRD) to end-stage kidney disease (ESKD) long time ago, however, authors are still using this old terminology. In the latest publication of KDIGO, they recommended clearly to use "kidney" rather than "renal" or "nephro-"* [*https://pubmed.ncbi.nlm.nih.gov/32409237/*](https://urldefense.proofpoint.com/v2/url?u=https-3A__pubmed.ncbi.nlm.nih.gov_32409237_&d=DwQGaQ&c=sJ6xIWYx-zLMB3EPkvcnVg&r=PwNLuw2JhLE3j9YZls-nRgEVrW5Zg71JsizODOOWGmtqGhCKdsvOOD5Ia41WUrp2&m=xn6mEks_xUAB_G32s-4EW4j7DVsdyYrA8J0l7x6gr8Tid4QOgmXUgrSDE2re6Wzm&s=WOz28DU94_3KyEAXvPbVwGogUavK6Ties1xvoDbZkCo&e=) *Is it appropriate to ask patients about term that will be recently changed to make it more clear for patients' understanding?*

We fully agree. The survey was developed during the ongoing debate in the renal community about patient-centered verbiage. Thus, at the time, we had used these terminologies to assess patient comfort with this verbiage. As for these two items in particular, during analysis we found that they both had high discriminatory ability, above our threshold of “excellent” at ≥.40 for the discrimination index, which encouraged us to retain the items. Interestingly, both items also had high proportions of correct responses—about 79% and 81% for items 2 and 15 respectively. Item 15, however, met our threshold for “too easy” with item difficulty score of .81, and thus we removed it from the reduced version of the scale. For future implementation of the survey, we will replace renal/nephron and implement “kidney” where possible and appropriate (and analyze additional data, as necessary, for item and scale validation). We thank the reviewer for raising this point and have discussed it under the limitations and future directions portion of the Discussion.

2. *Checking the reliability score from S2 item level analysis provided by the authors ... I find the reliability score are far below accepted standards worldwide. All scores are below 0.5 in the full scale, reduced scale total scores and subdomain scores! The authors didn't discuss these results in their manuscript or explain these low reliability score levels?*

We appreciate the reviewer’s examination of appendix tables!

While we agree that the item-level reliability scores may appear low initially, we assert that these values do not alone justify removal of the items or suggest that they are unacceptable. First, item-level reliability is not the same index as test reliability (e.g., Cronbach’s alpha), and so should be not judged by the same standards. Domain-level test reliability scores, as calculated by Cronbach’s alpha, are .59 for General Kidney Knowledge, .84 for CKD Knowledge, .83 for ESKD Knowledge. Cronbach’s alpha for the General Kidney Knowledge domain is lower than would be generally desired, but it should be noted that alpha is also a function of number of items, and so the small number of items in the General Kidney Knowledge Domain, considering the goal of developing an ESKD knowledge awareness tool is also contributing to the relatively low Cronbach’s alpha.

Overall, to draw attention to the distinction between item-level and test-level reliability scores, we have revised the Supplementary Materials to include Cronbach’s alpha for the domains and the full scale.

As for thresholds on *item-score* reliability, while some older work (Wanous *et al.*, 1997) has suggested a minimum of .70, more recent work (Zijlmans *et al.*, 2018) considers this cutoff to be too stringent and suggest a threshold of .30 for item-score reliability. For the reduced questionnaire, the number of items per domain meeting threshold is 3/5 for General Kidney Knowledge, 6/14 for CKD Knowledge, and 4/13 for ESKD Knowledge. However, it is universally agreed that there is still much to learn about the relationship between item-score reliability and test-score reliability, including the impact of removing low item-score reliability items (Zijlmans *et al.*, 2018).

All of this being said, item-level and test-level reliability scores reside amongst multiple metrics by which to judge items for measurement development, and individual item metric scores should be considered in this broader context. Future work will focus on further measure refinement, including factor analysis which could suggest additional items for domain recategorization, item revision, or item removal altogether.

3. *Please provide the reduced scale questionnaire including the 32 items. The reader will be interested in knowing which items were removed and what are the remaining items?*

We apologize for this omission and have provided a reduced version of the questionnaire to be included in the revision.

4. *The authors stated in their limitations sections that there are many shortcomings and that they are planning to address as many of these shortcomings as possible, however, in subsequent work. I understand and accept that the test be tested for correlation with hard outcomes in further studies. However, if there are additional steps for scale validation needed, why weren't they finished before publishing this manuscript? The authors stated this clearly in their limitations section: "Boateng et al. (2018) outlined nine steps for scale validation and development, and not all steps have been completed here."*

We appreciate the reviewer’s concern for completing all stages of a validation project prior to publication. Boateng et al. (2018) outlined a series of steps that represent an ideal situation, but they themselves note that the necessity of all nine steps will vary from study to study, and that researchers will have to make their own decisions about which steps to prioritize based on study goals, resource constraints, etc. We assert that not all steps can feasibly be reported in a single manuscript, nor should they be. For example, some scale development steps involve qualitative methodology such as focus groups and individual interviews, and we feel that such efforts deserve their own dedicated study and write-up. For the purposes of this study, we felt that a focus on the initial quantitative steps was warranted, both for manuscript parsimony and to make the scale more readily available to other researchers and clinicians who currently lack scale to evaluate ESKD awareness .

**Reviewer #2:** In the current manuscript, Orozco et al developed a questionnaire representing “patient disease awareness instrument”, as the first step of a multistep project. Initially, the questionnaire included 45 items distributed under 3 subdomains. Considering item difficulty and item discrimination index scores, it was then reduced to 32 items, (5/10 items in the general kidney knowledge subdomain, 14/21 items in the CKD knowledge subdomain, and 13/14 items in the ESKD knowledge subdomain). Below are my comments:

1. *The manuscript focused only on the development of the survey as an initial step of a multistep project. However, authors stated the purpose of the project as a whole and did not specifically address the aim of the manuscript, which can be confusing for the reader (page /9/23; last paragraph).*

We agree with the reviewer that we did not clearly communicate the overarching vs current manuscript goals. The overall goal of this project is to prospectively evaluate the effects of patients’ awareness of kidney disease and their health-related quality of life on patients’ overall health outcomes and renal replacement therapy selection. To achieve this, the baseline data included evaluation of the patients’ awareness of kidney disease. However, considering that we do not have a validated questionnaire for evaluating ESKD awareness, the first step of the project focused on validating a multi-step ESKD questionnaire. We have updated the manuscript to clarify these goals (specifically, we have updated the final paragraph of the Introduction section and the first paragraph of the Discussion section to clarify the specific study goals, rather than just the overarching goal).

2. *The item difficulty index, if I understand well, was calculated as c/n, where c the number of correct answers to a question and n the total number of respondents to that question i.e., the percent of correct answers (page 11/23).*

The reviewer is correct, and we thank them for pointing out this egregious miscommunication; it has been corrected in the manuscript.

3. *Appendix 1 included all the 45 items. I suggest identifying the removed items and distributing the questions under the 3 subdomains.*

Thank you, this comment pertains to the similar issue raised by the Reviewer 1 in #3 above. We are providing the full survey with their domains, with the removed items marked off in the supplemental material.

4. *I suggest it would be more interesting to define participant demographics and characteristics.*

We thank the reviewer for this suggestion; we have included participant demographics and characteristics in the beginning of the Results section of the manuscript.

5. The practicality of using a relatively long survey of 32 MCQ items in every day clinical practice need to be discussed.

We agree with the reviewer that a discussion on survey length and practicality was missing, and we have therefore updated the Discussion section of the manuscript to address it (see paragraph 9 of the Discussion section).

WANOUS, J. P.; REICHERS, A. E.; HUDY, M. J. Overall job satisfaction: How good are single-item measures? **Journal of Applied Psychology,** v. 82, n. 2, p. 247-252, 1997. ISSN 1939-1854(Electronic),0021-9010(Print).

ZIJLMANS, E. A. O. et al. Item-Score Reliability in Empirical-Data Sets and Its Relationship With Other Item Indices. **Educational and Psychological Measurement,** v. 78, n. 6, p. 998-1020, 2018. Available at: < <https://journals.sagepub.com/doi/abs/10.1177/0013164417728358> >.
